# Association of vitamin D deficiency with insulin resistance among type 2 diabetes mellitus patients - A case-control study

**DOI:** 10.6026/973206300212897

**Published:** 2025-08-31

**Authors:** Pankaj Kumar Jain, Preeti Nigotia, Arun Mishra, Lalan Pratap Singh

**Affiliations:** 1 Department of General Medicine, NSC Government Medical College, Khandwa, Madhya Pradesh, India; 2 Department of General Medicine, SRVS Medical College, Shivpuri, Madhya Pradesh, India; 3 Department of Biochemistry, NSC Government Medical College Khandwa, Madhya Pradesh, India; 4 Department of General Medicine, Government Medical College, Satna, Madhya Pradesh, India

**Keywords:** Vitamin D deficiency, type 2 diabetes mellitus, insulin resistance, HOMA-IR, case-control study

## Abstract

The association between vitamin D deficiency and insulin resistance is of interest. Hence, 140 participants-70 type 2 diabetes mellitus (T2DM) patients and 70 age- and sex-matched healthy controls were evaluated. Serum 25(OH) vitamin D levels and HOMA-IR scores were measured and compared. Vitamin D levels were significantly lower and HOMA-IR significantly higher in T2DM patients compared to controls. An inverse correlation was observed between vitamin D levels and insulin resistance. Thus, we show a potential role of vitamin D deficiency in the pathophysiology of insulin resistance in T2DM.

## Background:

Type 2 diabetes mellitus (T2DM), a chronic metabolic disorder characterized by insulin resistance and relative insulin deficiency [1]. It is a major public health concern globally, contributing to significant morbidity and mortality [2]. While the pathogenesis of T2DM is multifactorial, emerging evidence has identified a possible link between micronutrient deficiencies-particularly vitamin D deficiency and insulin resistance [3]. Vitamin D is known to play a role in glucose metabolism through its effects on pancreatic β-cell function and insulin sensitivity [4]. Hypovitaminosis D has been widely reported in individuals with T2DM, but whether this relationship is causal or associative remains debated [5]. Several observational studies have suggested that lower levels of 25-hydroxyvitamin D [25(OH) D] may be associated with increased insulin resistance [6]. The vitamin D-endocrine system plays a major role in physiological processes that modulate mineral metabolism and immune function with probable link to several chronic and infectious conditions [7]. Therefore, it is of interest to evaluate the association between serum vitamin D levels and insulin resistance in patients with T2DM through a case-control design, comparing them with age- and sex-matched non-diabetic individuals.

## Materials and Methods:

This case-control study was conducted at the Department of Endocrinology of a tertiary care teaching hospital between January 2023 and December 2023. A total of 140 participants were enrolled, comprising 70 patients diagnosed with type 2 diabetes mellitus (T2DM) and 70 age- and sex-matched healthy controls. The T2DM group included individuals aged between 30 and 65 years who had been diagnosed with diabetes for at least one year, based on ADA criteria. The control group consisted of healthy individuals with no history of diabetes, metabolic syndrome, or any chronic systemic illness. Participants with chronic kidney disease, hepatic disorders, malabsorption syndromes, endocrine abnormalities other than diabetes, or those who were on vitamin D supplementation or medications affecting glucose metabolism (other than anti-diabetic agents in cases) were excluded. After obtaining informed consent, fasting venous blood samples were collected from all participants for biochemical analysis. Serum 25-hydroxyvitamin D [25(OH)D] levels were measured using chemiluminescence immunoassay. Vitamin D deficiency was defined as serum 25(OH)D levels below 20 ng/mL. Data were statistically analyzed using SPSS version 26. Continuous variables were expressed as mean ± standard deviation and compared using the unpaired t-test. Pearson's correlation coefficient was used to analyze the relationship between vitamin D levels and HOMA-IR, with a p-value of less than 0.05 considered statistically significant.

## Results:

A total of 140 participants were analyzed, including 70 T2DM patients and 70 healthy controls. The two groups were comparable in age and gender distribution. T2DM patients showed significantly lower serum 25(OH) vitamin D levels and higher HOMA-IR values, indicating a clear association between vitamin D deficiency and insulin resistance. An inverse correlation between vitamin D levels and HOMA-IR was observed in the diabetic group. Table 1 (see PDF) shows the age and gender distribution between the T2DM and control groups were statistically similar, eliminating demographic bias. Table 2 (see PDF) shows Mean serum vitamin D levels were significantly lower in T2DM patients compared to controls. Table 3 (see PDF) shows the prevalence of vitamin D deficiency was markedly higher in the diabetic group. Table 4 (see PDF) shows Fasting insulin levels were significantly elevated in the T2DM group, reflecting increased insulin resistance. Table 5 (see PDF) shows Fasting blood glucose levels were significantly higher in diabetic patients, consistent with their diagnosis. Table 6 (see PDF) shows HOMA-IR values were significantly higher in the T2DM group, confirming greater insulin resistance. Table 7 (see PDF) shows an inverse correlation was observed between serum vitamin D levels and HOMA-IR in the diabetic group. Table 8 (see PDF) shows Vitamin D deficiency was associated with significantly higher insulin resistance in diabetic patients. Table 9 (see PDF) shows a larger proportion of vitamin D-deficient diabetics had poorly controlled glucose levels. Table 10 (see PDF) shows Body Mass Index (BMI) was slightly higher in T2DM patients but not statistically significant between groups.

## Discussion:

This case-control study demonstrates a significant association between vitamin D deficiency and insulin resistance among patients with type 2 diabetes mellitus (T2DM). The findings reveal that T2DM patients had markedly lower serum 25(OH) vitamin D levels and significantly higher HOMA-IR scores compared to age- and sex-matched healthy controls. This supports previous evidence suggesting that vitamin D may play an essential role in maintaining insulin sensitivity and glucose metabolism [8]. The inverse correlation found between vitamin D levels and insulin resistance in the diabetic group reinforces the hypothesis that vitamin D deficiency contributes to the pathophysiology of insulin resistance [9]. Vitamin D is believed to enhance insulin receptor expression and improve insulin responsiveness in peripheral tissues. It may also influence calcium homeostasis in pancreatic β-cells, affecting insulin secretion [10]. Deficiency in vitamin D might, therefore, impair both insulin production and its action, contributing to the development and worsening of T2DM [11]. Furthermore, the significantly higher prevalence of vitamin D deficiency in T2DM patients underscores the need for routine screening and potential correction of this modifiable risk factor. Diabetics with vitamin D deficiency also had poorer glycemic control, as indicated by higher HbA1c levels, which could suggest an added burden on disease management [12]. Although BMI was marginally higher in the diabetic group, it was not statistically significant, ruling out obesity as a major confounder in this cohort. Despite the compelling associations observed, this study has limitations. Being observational and retrospective, it cannot establish a causal relationship [13]. Other factors such as sun exposure, dietary intake and physical activity were not controlled, which might have influenced vitamin D levels. Nevertheless, the consistency of findings with existing literature suggests that vitamin D status should not be overlooked in the clinical management of diabetes [14, 15]. These results support the potential role of vitamin D supplementation as an adjunct in managing insulin resistance and improving metabolic control in T2DM patients. Future large-scale randomized controlled trials are necessary to determine whether vitamin D supplementation can lead to meaningful improvements in insulin sensitivity and long-term diabetic outcomes.

## Conclusion:

A strong inverse association between vitamin D deficiency and insulin resistance in patients with type 2 diabetes mellitus. Diabetic individuals with lower vitamin D levels exhibited significantly higher HOMA-IR scores and poorer glycemic control. These findings emphasize the potential role of vitamin D in glucose metabolism and support the consideration of routine vitamin D assessment and correction as part of comprehensive diabetes management.

## References

[1] Cavalier E (2011). Journal of Diabetes Metab..

[2] Aludwan M (2020). Journal of Minerva Endocrinol..

[3] Wimalawansa SJ (2016). J Steroid Biochem Mol Biol..

[4] Gharekhani A (2020). Journal of Pharm Res..

[5] Alhumaidi M (2013). Journal of Maedica (Bucur)..

[6] Dhas Y (2019). Journal of Clin Chim Acta..

[7] Fondjo LA (2017). PLoS One..

[8] Chen S (2016). Journal of Bone Miner Res..

[9] Pilz S (2013). Journal of Curr Diab Rep..

[10] Calvo-Romero JM (2015). Journal of Investig Med..

[11] Pan GT (2016). Journal of Nutr Sci Vitaminol (Tokyo)..

[12] Zhang J (2016). Journal of Endocrinol..

[13] Ryu OH (2014). Journal of Intern Med..

[14] Al-Shoumer KA (2013). Journal of Prim Care Diabetes..

[15] Dutta D (2013). Journal of Med Res..

